# Design of an Event-Driven, Low-Power Wearable Fall Detection Device for Hospital Use in Older Adults: A Proof-of-Concept

**DOI:** 10.7759/cureus.103024

**Published:** 2026-02-05

**Authors:** Endrit Hasani

**Affiliations:** 1 Emergency Medicine, Independent Researcher, Medical Device Engineering, Prishtina, ALB

**Keywords:** bluetooth low energy, event-driven sensing, fall detection, healthcare technology, hospital safety, inpatient monitoring, low-power electronics, older adults, patient safety, wearable device

## Abstract

As life expectancy increases, a growing proportion of hospitalized older adults require continuous medical supervision during inpatient care. Falls among hospitalized older adult inpatients are a major patient-safety challenge, associated with increased injury rates, prolonged hospital stays, and higher healthcare costs. Adoption of existing fall detection solutions has been limited by patient privacy concerns, high implementation costs, inconsistent detection performance, such as false alarms, limited battery life, and discomfort associated with intrusive wearable designs.

This study presents a compact and lightweight wearable device for fall detection and alerting designed for hospital environments. Bluetooth Low Energy connectivity enables transmission of alerts to a paired mobile device or nurse station gateway used by healthcare staff, while an onboard audible buzzer provides immediate notification to nearby personnel within the patient room. The device is battery-powered and rechargeable, enabling extended operation without frequent recharging.

The wearable fall detection device was successfully implemented as a compact, clip-on system integrating inertial sensing, local alerting, and Bluetooth Low Energy communication. An event-driven architecture enabled continuous motion monitoring with low average power consumption, resulting in an estimated battery life of approximately 1,800 hours under idle conditions and 100-200 hours under a defined worst-case continuous activity scenario, based on component datasheet current consumption and assumed duty cycles. The system supports both immediate audible alerts for nearby staff and remote notifications via Bluetooth, enabling timely intervention in hospital settings*.*

The proposed wearable device monitors patient movement using an integrated accelerometer to identify motion patterns consistent with fall-like events, based on predefined acceleration-based trigger conditions. In combination with Bluetooth-based communication, the system enables real-time interaction with external devices. Powered by a rechargeable battery designed for long-lasting operation, the device represents a practical solution to the identified patient safety challenge.

The device was evaluated at the bench and system-integration level through functional testing of motion-triggered event detection, alert generation, and wireless communication. Performance evaluation focused on system responsiveness, power consumption profiles, and estimated battery life under defined operating scenarios rather than clinical outcome validation. No human subject testing was conducted, and results reflect a proof-of-concept implementation intended to inform future validation studies.

## Introduction

Rising emergency department (ED) volumes contribute to downstream inpatient crowding and workflow strain, which can limit staff availability and timely patient monitoring in hospital settings [1]. Prior research has demonstrated that a rapidly aging population and the complex care needs of older adults are associated with increased emergency department utilization and crowding, placing substantial strain on ED operations and resources [2]. Older adults are more likely to experience declines in physical function, balance, and mobility, which contribute to a higher incidence of falls and fall-related injuries [3,4].

In the United States, falls are highly prevalent among older adults, with over 14 million individuals aged 65 years and older reporting at least one fall annually, highlighting the vulnerability of this population that is also heavily represented in hospital inpatient settings [5] and about 37% of those who fall reported an injury that required medical treatment or restricted their activity for at least one day, resulting in an estimated nine million fall injuries [6].

In hospital environments, a substantial proportion of inpatient falls occur during transitions such as getting out of bed or other mobility activities, and many fall events happen when patients are not observed [7]. Older adults are at higher risk for falls due to intrinsic factors, including reduced mobility, balance impairment, and cognitive dysfunction such as delirium or dementia [8]. Hospital falls are strongly associated with fractures, head injuries, prolonged length of stay, increased readmission rates, and higher healthcare costs, thereby further exacerbating the burden on emergency and inpatient services [7]. Despite the widespread implementation of fall-prevention protocols, current inpatient fall-prevention approaches rely heavily on staff observation, physical restraints, and basic bed-exit alarms, which typically trigger only after patient movement has begun and have been associated with high false-alarm rates, contributing to alarm fatigue among healthcare personnel [9].

These limitations underscore the need for unobtrusive, reliable, and timely fall detection and prevention systems specifically designed for hospital bed settings.

Wearable sensing technologies offer a promising alternative to traditional hospital fall-prevention methods by enabling continuous, patient-specific monitoring without reliance on constant staff observation or environmental alarms. By capturing motion and posture changes in real time, wearable systems can promptly detect fall-related events and trigger alerts to support timely staff response. When designed for comfort and compliance, such devices have the potential to reduce false alarms, limit alarm fatigue, and ease the burden on healthcare personnel.

Despite their promise, wearable systems face notable limitations, including restricted battery life, discomfort that reduces user adherence, variable clinical accuracy, and commonly cited concerns related to data privacy and security, which must be addressed at the system design and deployment level [10-12].

To address these limitations, this paper presents the design of a compact and lightweight wearable device that can be clipped onto a patient’s clothing for continuous movement monitoring. Owing to its low mass and unobtrusive form factor, the device is unlikely to cause discomfort or interfere with normal patient activity. An integrated motion sensor detects potentially unsafe movements and provides immediate alerts to nearby staff via an onboard buzzer, while Bluetooth connectivity enables remote notifications to healthcare personnel at a distance. The system is powered by a rechargeable battery and designed for practical use in hospital environments.

## Technical report

The device is designed as a lightweight clip-on module intended to be attached to the patient’s clothing near the upper chest region, selected as a practical and unobtrusive placement for inpatient use. This placement minimizes discomfort and avoids issues associated with adhesive or body-mounted wearables, which is particularly important for older adults and patients with sensitive skin. However, the impact of placement location and clothing variability on signal quality and detection performance requires further validation. In combination with inertial sensing using an accelerometer and gyroscope, the clip-on form factor enables reliable detection of gross body movements while maintaining patient comfort and supporting prolonged use in hospital environments.

**Figure 1:**
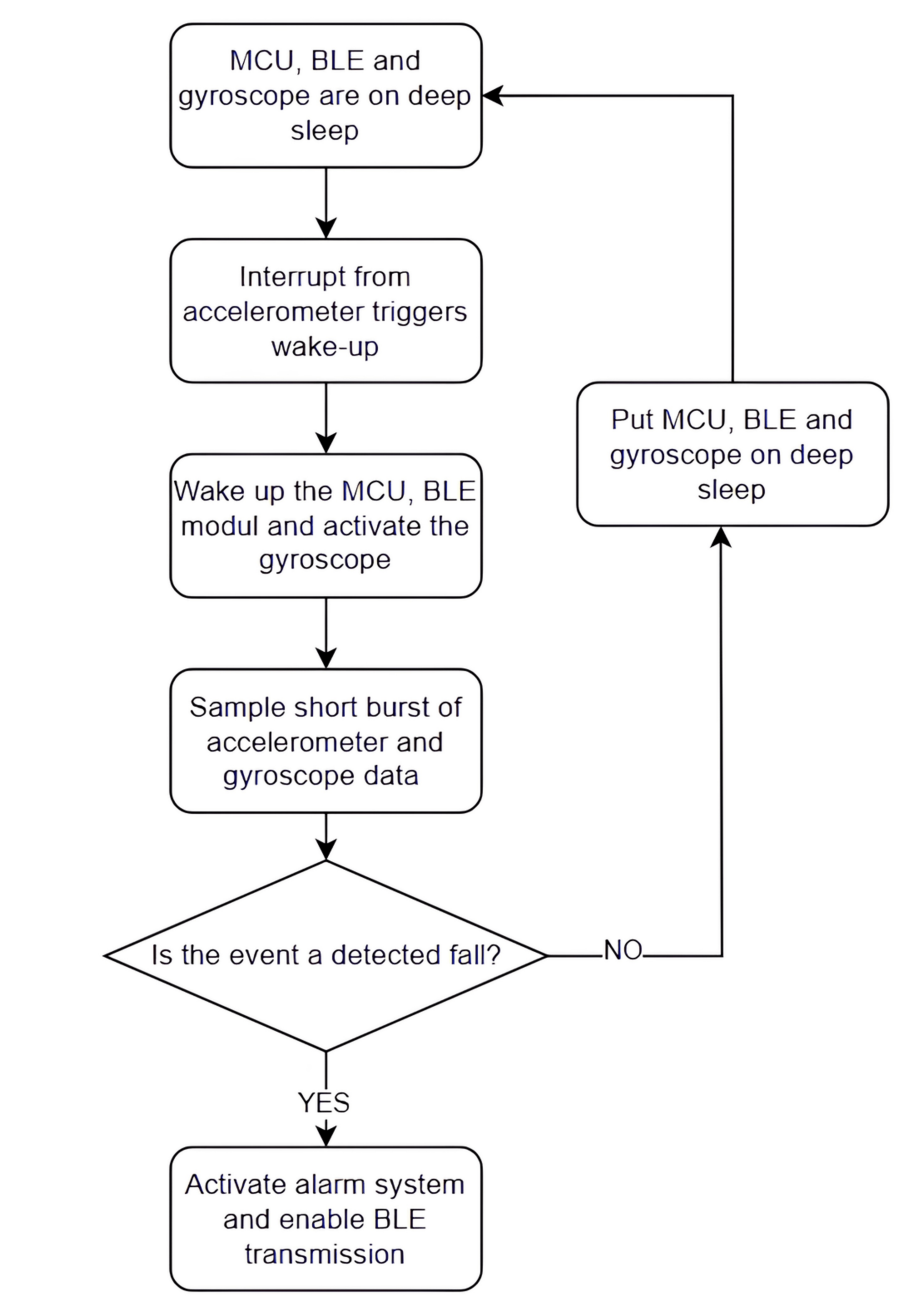
Low-Power Event-Driven Fall Detection Device Workflow Fall event classification is performed using a lightweight rule-based approach applied to a short burst of inertial data following the accelerometer interrupt. Features include acceleration magnitude, abrupt changes in orientation, and brief post-event inactivity. Events exceeding predefined acceleration and duration thresholds are classified as fall-related, while transient movements below these thresholds are discarded to reduce false triggers. Sampling frequency and burst duration were selected to balance detection reliability and power consumption. MCU - microcontroller unit; BLE - Bluetooth Low Energy.

In the default state, the microcontroller unit (MCU), Bluetooth Low Energy (BLE) module, and gyroscope remain in deep-sleep mode to minimize power consumption, while the accelerometer operates in an ultra-low-power interrupt mode. When the accelerometer detects a motion pattern exceeding predefined thresholds, it generates an interrupt that wakes the MCU and enables subsequent system components. The system then samples a short burst of accelerometer and gyroscope data, which is analyzed to determine whether the detected event corresponds to a fall. If the event is classified as non-critical, all components return to deep sleep. If a fall is confirmed, the alarm system is activated, and BLE transmission is enabled to notify caregivers or monitoring systems, after which the system can return to its low-power state as shown in Figure 1.

The NINA-B306 module (u-blox AG, Thalwil, Switzerland) serves as the main control unit of the device, providing a low-power Bluetooth-enabled microcontroller responsible for coordinating sensor data acquisition, local processing, and wireless communication with external devices. The module integrates Bluetooth Low Energy (BLE) functionality in a compact form factor and supports firmware updates via controlled wired programming or restricted BLE-based update modes, with updates disabled during normal clinical operation [13].

The LIS2DW12TR accelerometer (STMicroelectronics, Geneva, Switzerland) is employed as an always-on, ultra-low-power motion sensor responsible for continuous monitoring of patient movement. Hardware-based activity, inactivity, and free-fall detection features are configured using predefined acceleration thresholds and duration windows (on the order of ~1.5-2 g for activity detection and brief inactivity periods following high-acceleration events), enabling autonomous interrupt generation without continuous microcontroller involvement. This configuration allows the system to remain in deep sleep during normal conditions while reliably triggering wake-up on fall-like motion patterns.

The LSM6DSOXTR inertial measurement unit (STMicroelectronics, Geneva, Switzerland) is activated following a trigger event to perform short-duration, high-fidelity motion analysis. Gyroscope and accelerometer data are sampled at a moderate rate (e.g., 50-100 Hz) over a brief time window to capture angular velocity and orientation changes, which are used to distinguish abrupt fall-like rotations from normal voluntary movements. By combining a three-axis accelerometer with a three-axis gyroscope, the device enables short-duration sampling of linear acceleration and angular velocity to verify and classify suspected fall events. The gyroscope is activated only during brief confirmation windows, ensuring improved motion characterization while minimizing additional power consumption.

**Figure 2:**
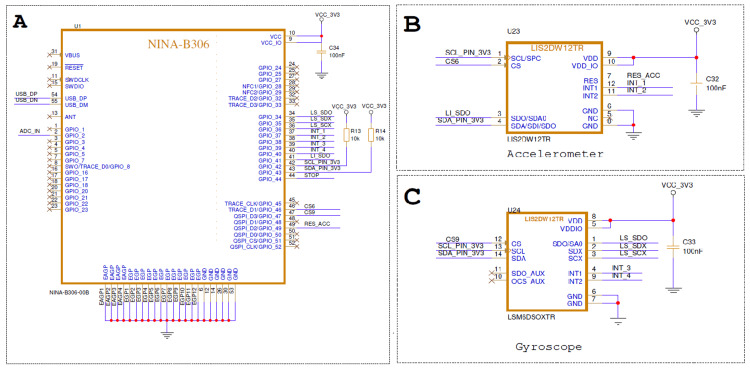
System Schematic of the Wearable Fall Detection Device (A): System schematic showing the NINA-B306 Bluetooth Low Energy microcontroller; (B): LIS2DW12TR ultra-low-power accelerometer; (C): LSM6DSOXTR inertial measurement unit with integrated gyroscope used for event-driven fall detection.

Figure 2 illustrates the core electronic architecture of the proposed fall detection device. The NINA-B306 Bluetooth Low Energy module functions as the central processing and communication unit, interfacing with the inertial sensors and managing system power states. An ultra-low-power LIS2DW12TR accelerometer operates continuously to monitor patient motion and generate hardware interrupts upon detection of predefined movement thresholds, enabling event-driven wake-up of the system. Upon activation, the LSM6DSOXTR inertial measurement unit is temporarily enabled to acquire higher-resolution acceleration and angular velocity data for confirmation and classification of suspected fall events. All components are powered from a regulated 3.3 V supply with local decoupling to ensure stable operation, supporting a low-power design that balances continuous monitoring with extended battery lifetime.

Table 1 provides a high-level overview of the estimated energy consumption of the proposed wearable system during typical hospital use. Ultra-low-power components, such as the always-on accelerometer and the microcontroller in deep-sleep mode, dominate system operation, while higher-current elements, including the gyroscope, Bluetooth transmission, and audible alarm, are activated only during short, event-driven intervals. By minimizing active-duty operation and relying on interrupt-based sensing, the system achieves low average current consumption, supporting extended battery life while maintaining timely alerting capabilities. All values represent estimated typical currents based on manufacturer datasheets and are intended to contextualize battery life expectations rather than report measured clinical performance.

**Table 1:** Estimated Power Consumption and Duty-Cycle-Based Average Current of the Proposed Wearable System Summary of the primary system subsystems, their operating modes, typical current consumption, estimated duty cycles, and resulting average current contribution. Values are derived from component datasheet specifications and reflect expected behavior under typical inpatient monitoring conditions. MCU - microcontroller unit; BLE - Bluetooth Low Energy.

Subsystem	Mode	Typical Current (µA/mA)	Duty Cycle	Average Current
Accelerometer (LIS2DW12TR)	Always-on low-power	~1–2 µA	~100%	~1–2 µA
MCU + BLE (NINA-B306)	Deep sleep	~3–5 µA	~99%	~3–5 µA
MCU + BLE	Active + BLE TX	~5–10 mA	<1%	~50–100 µA
Gyroscope (LSM6DSOXTR)	Event-triggered	~0.6–1 mA	Rare	~5–10 µA
Buzzer	Alarm active	~20–30 mA	Very rare	Negligible

**Figure 3:**
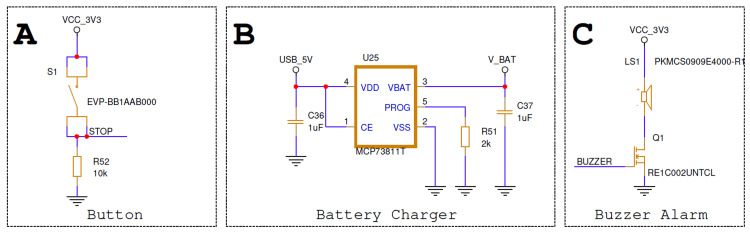
Power Management, User Interaction, and Local Alerting Subsystems (A): User-accessible control button allowing temporary suspension of motion detection during supervised patient activity. (B): Lithium-ion battery charging circuitry used for safe recharging from a universal serial bus (USB) power source. (C): Audible buzzer circuit providing local alerts to nearby healthcare staff.

Figure 3 illustrates the supporting subsystems responsible for power management, user interaction, and local alerting. A dedicated lithium-ion battery charger enables safe and controlled charging from a universal serial bus (USB) power source, allowing routine recharging without device removal. A low-power buzzer driven by a transistor switch provides an immediate audible alert to nearby healthcare staff when a fall event is confirmed, enabling rapid on-site response without reliance on wireless connectivity. In addition, a user-accessible push button is incorporated to temporarily suspend motion detection when the patient is intentionally assisted by healthcare personnel or requires supervised movement. This manual override helps prevent false alarms during routine care activities and supports practical integration of the device into clinical workflows. To reduce accidental or inappropriate deactivation, the manual override requires a deliberate long-press gesture (e.g., ≥3 seconds) to silence the local audible alarm. The override affects only the buzzer output and does not disable fall detection, data logging, or Bluetooth alert transmission. The override state is automatically cleared after a predefined timeout, after which normal alerting behavior resumes.

**Figure 4:**
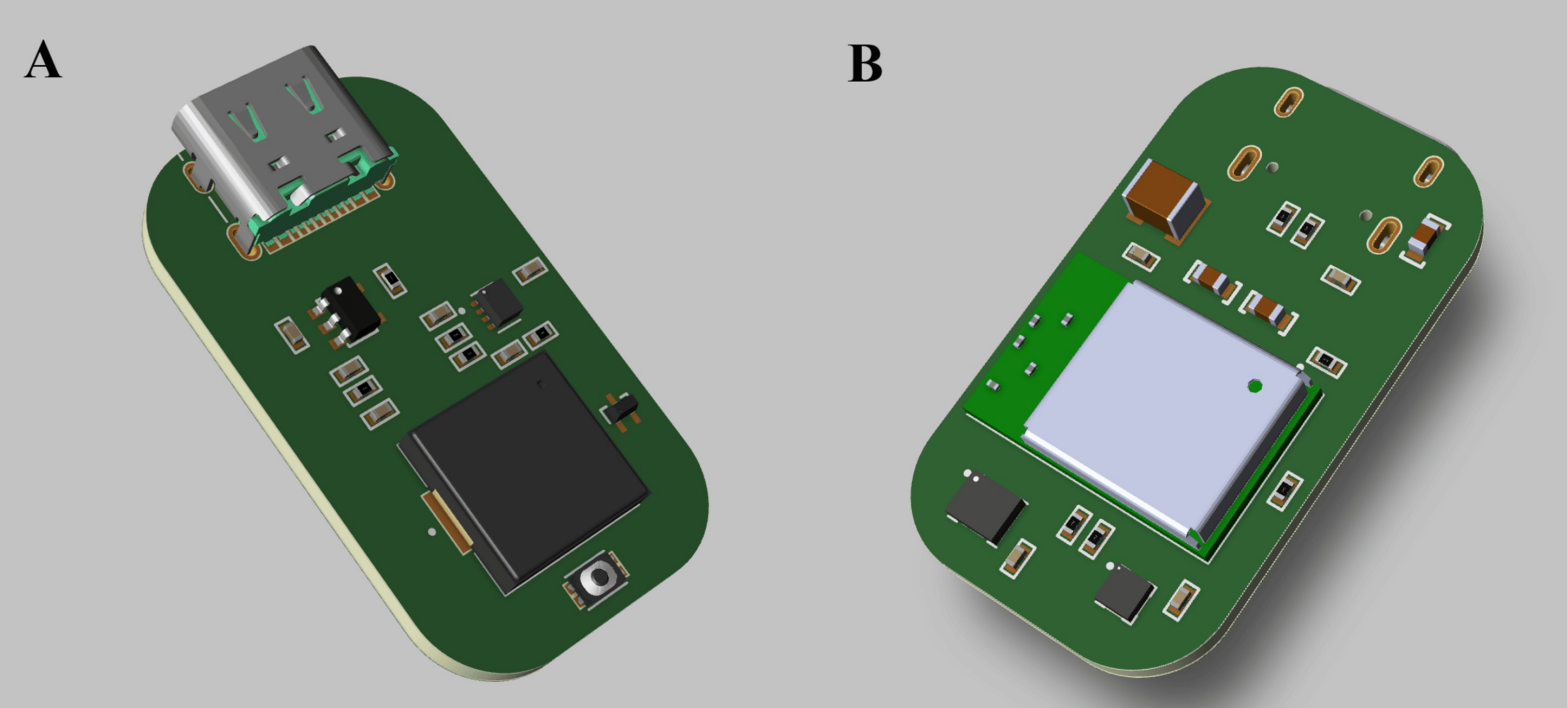
Three-Dimensional PCB Layout of the Wearable Fall Detection Device The PCB footprint is 1.8 × 3.1 cm, and the total device mass is expected to be only a few grams (prototype-dependent). The enclosure is intended for fabrication in a disinfectant-compatible polymer [e.g., acrylonitrile butadiene styrene (ABS)/ polycarbonate (PC) blend], with material selection to be validated against routine hospital cleaning agents. (A): top three-dimensional view; (B): bottom three-dimensional view, PCB - printed circuit board.

Figure 4 shows the three-dimensional printed circuit board layout of the proposed device, highlighting the placement of the main control unit, sensors, and power-management components. View A illustrates the top side of the printed circuit board (PCB), which hosts the USB charging interface, battery management circuitry, and the main processing and communication module. View B shows the opposite side of the board, where the inertial sensors and supporting passive components are placed to achieve a compact and balanced layout. The dual-sided design enables efficient use of board area while maintaining short signal paths, adequate power decoupling, and a low-profile form factor suitable for clip-on attachment to patient clothing.

**Figure 5:**
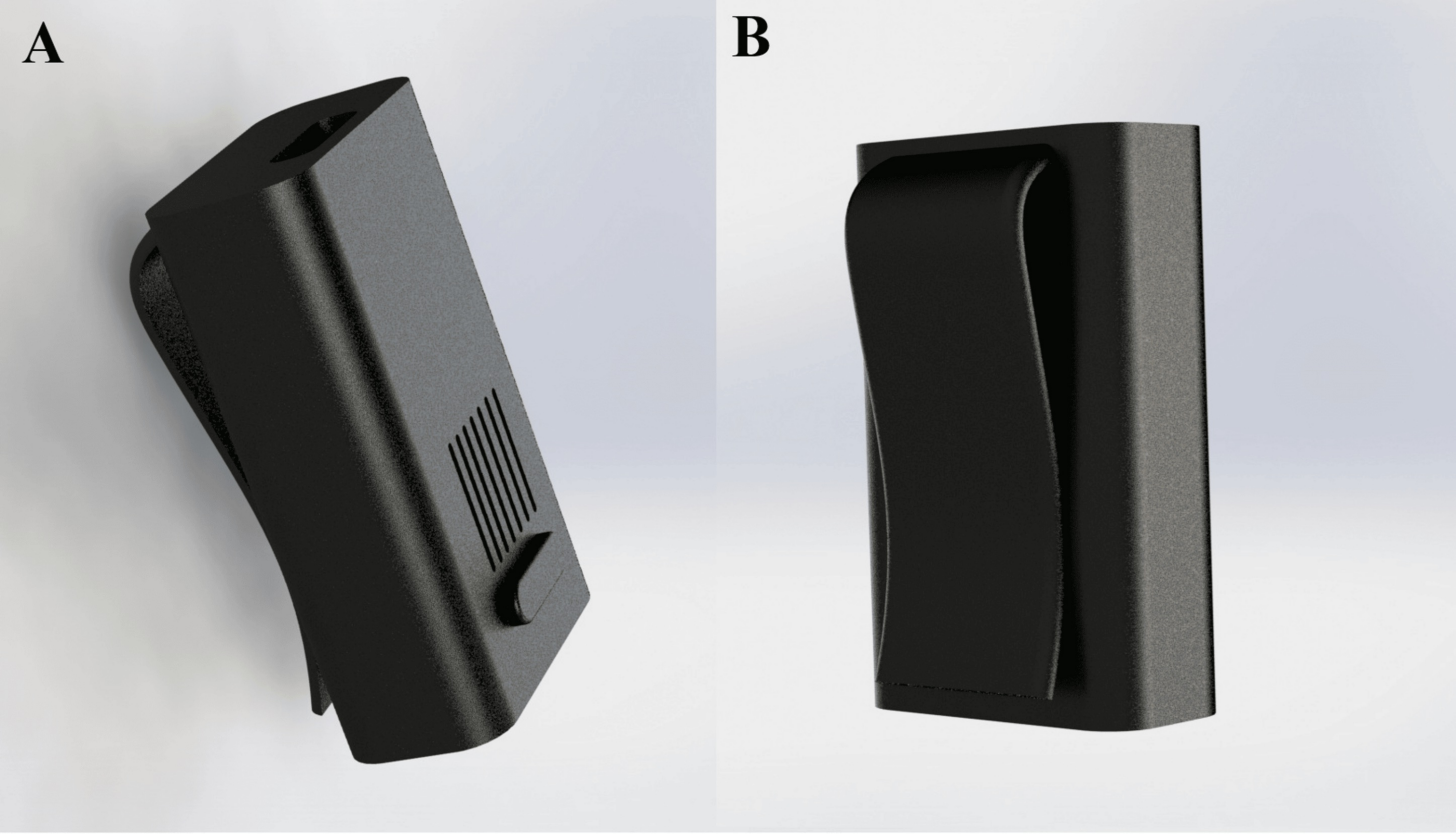
Enclosure Design and Clip-on Form Factor of the Wearable Device The enclosure is a conceptual design intended for fabrication in a rigid polymer [e.g., acrylonitrile butadiene styrene (ABS) or polycarbonate (PC)]; ingress protection and resistance to hospital-grade disinfectants have not yet been experimentally evaluated and will be addressed during future prototyping and validation. (A): front view; (B): back view.

Figure 5 illustrates the external enclosure design of the device, including its compact form factor and geometry intended for clip-on use. The enclosure supports secure retention of internal components while maintaining a smooth, sealed exterior suitable for routine cleaning and disinfection in hospital settings. Dedicated acoustic openings allow effective sound output from the integrated buzzer, and a user-accessible button is incorporated to enable temporary system suspension during supervised patient movement or clinical interventions. These design elements support reliable operation while accommodating practical workflow requirements in healthcare environments.

**Figure 6:**
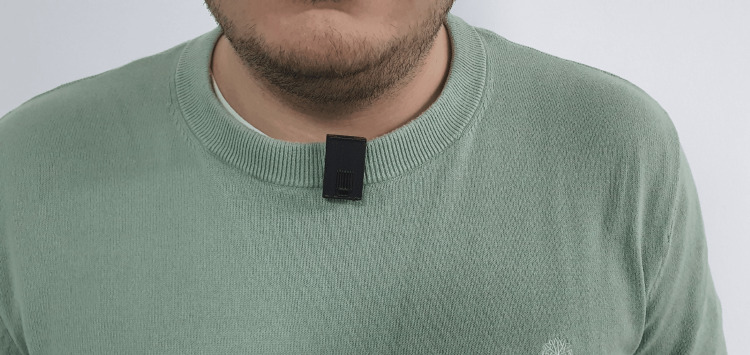
Demonstration of the Intended Wearing Position of the Wearable Device

Figure 6 demonstrates the intended wearing position of the device when clipped to the patient’s clothing near the upper torso without direct skin contact; however, placement performance may vary depending on garment type, fit, and patient movement characteristics. Chest-level placement was selected as a practical design choice for this proof-of-concept due to ease of use and reduced skin contact. However, clip stability and signal quality may vary across clothing types (e.g., gowns versus fitted garments) and patient behavior. Future iterations will evaluate alternative attachment methods and multi-location placement strategies to improve robustness across diverse inpatient populations.

Battery lifetime was estimated based on the system’s event-driven low-power architecture and typical operating modes of the selected components. During normal operation, only the LIS2DW12TR accelerometer remains active in an ultra-low-power mode (approximately 10-50 µA), while the NINA-B306 microcontroller and wireless interface remain in deep sleep (approximately 2-5 µA), resulting in a baseline current consumption of approximately 12-55 µA. Assuming a nominal 100 mAh lithium-ion battery and ideal voltage regulation, the estimated operating lifetime can be approximated using $t\,[\mathrm{h}] = \frac{C\,[\mathrm{mAh}]}{I\,[\mathrm{mA}]}$ yielding a range of approximately 1,800-8,300 hours, depending on the average current draw with longer lifetimes achievable under ideal low-activity conditions. High-power operations, including gyroscope activation and Bluetooth transmission, occur only briefly following rare trigger events and therefore contribute minimally to overall energy consumption. In a conservative worst-case illustrative scenario, defined as repeated motion triggers occurring every 10-20 seconds with sustained Bluetooth Low Energy transmission and brief audible alarm activation, the average current consumption is estimated to increase to approximately 0.5-1.0 mA, corresponding to an operating lifetime of approximately 100-200 hours. These estimates demonstrate that the proposed design can support operating lifetimes ranging from several days to multiple weeks, depending on usage patterns and system configuration.

## Discussion

Falls among hospitalized older adults remain a significant patient-safety concern and are associated with increased injury risk, prolonged hospital length of stay, and higher healthcare costs [2,5,6]. Existing fall-prevention approaches, including bed-exit alarms, staff observation, and environmental interventions, often suffer from delayed detection and alarm fatigue, limiting their effectiveness in busy clinical environments [4,7]. Wearable and sensor-based solutions have been proposed to address these limitations, but many rely on continuous sensing and wireless transmission, leading to reduced battery life, increased maintenance burden, and challenges with patient adherence [8-10].

The proposed device addresses these gaps through an event-driven, low-power wearable design that emphasizes simplicity of use and clinical practicality as guiding design considerations. By employing an always-on ultra-low-power accelerometer for initial motion detection and activating higher-power sensing and wireless communication only when necessary, the system supports extended battery life while maintaining timely alerting. The clip-on form factor avoids direct skin contact and enables rapid deployment without complex setup, addressing common usability barriers reported in wearable health monitoring systems [8,10]. While this work is limited to a proof-of-concept evaluation and requires further clinical validation, it demonstrates a feasible and scalable approach to inpatient fall detection that aligns with hospital workflow constraints and the growing need for patient-centered safety technologies [1,4,7].

## Conclusions

This study presented the design and implementation of a compact, low-power wearable device for fall detection in hospital environments, specifically targeting the needs of hospitalized older adults. By combining an event-driven sensing architecture with local audible alerting and Bluetooth-based remote notification, the proposed system is designed to support timely alerting while minimizing power consumption and maintenance requirements. The clip-on form factor and rechargeable design are intended to facilitate unobtrusive use and practical integration. While the current work is limited to a proof-of-concept evaluation, the results demonstrate the feasibility of an energy-efficient and user-centered approach to inpatient fall detection, warranting staged future evaluation, including bench testing, simulated validation, and pilot clinical studies.
